# Elevated Alcohol Consumption and Chronic Inflammation Predict Cardiovascular Risk Among Black Americans: Examination of a Dual-Risk Model Using Epigenetic Risk Markers

**DOI:** 10.3390/epigenomes9040040

**Published:** 2025-10-07

**Authors:** Steven R. H. Beach, Robert A. Philibert, Mei-Ling Ong, Man-Kit Lei, Kaixiong Ye

**Affiliations:** 1 Center for Family Research, The University of Georgia, Athens, GA 30602, USA; 2 Department of Psychology, The University of Georgia, Athens, GA 30602, USA; 3 College of Medicine, University of Iowa, Iowa City, IA 52242, USA; 4 Department of Sociology, The University of Georgia, Athens, GA 30602, USA; 5 Department of Genetics, The University of Georgia, Athens, GA 30602, USA

**Keywords:** alcohol consumption, inflammation, aging, cardiac risk

## Abstract

Background: Heart disease may take a greater toll on Black Americans than White Americans despite similar levels of traditional risk factors. Elevated alcohol consumption (EAC) and chronic inflammation are two potentially important additional risk factors to consider. Both are relevant to understanding health disparities in cardiovascular health. Methods: Couples with a Black preadolescent or early adolescent child living in the home were recruited and followed. In waves 5 and 6 of data collection, biological samples were also collected allowing the characterization of elevated alcohol consumption, chronic inflammation, and cardiac risk using DNA methylation indices. 383 individual partners comprising 221 couples were examined across the two waves of data, yielding 661 person-wave observations from 383 individuals. Results: EAC at wave 5 forecast increased cardiac risk at W6 (R^2^ change = 0.276), β = −0.193, *p* = 0.001. However, chronic inflammation at wave 5 did not add significantly to the baseline model, β = −0.042, *p* = 0.549. Conversely, the slope of change for chronic inflammation was associated with slope of change in cardiac risk (R^2^ change = 0.111), b = −0.014, *p* = <0.001, but EAC change was not significantly associated with change in cardiac risk, b = −0.001, *p* = 0.185. Conclusions: Elevated alcohol consumption may be an important risk factor for increased cardiac risk over time in middle age. If so, it could be an important avenue for preventative intervention to decrease cardiac risk. Future research should examine whether similar associations are observed for other racial or minoritized groups and for non-minoritized groups.

## 1. Introduction

Heart disease has been the leading cause of death in the United States since the 1950s. Black Americans have consistently shown poorer cardiovascular health than non-Hispanic White Americans and experience higher rates of CVD mortality [1,2,3]. Despite advances in diagnosing, preventing, and managing cardiovascular disease, substantial race-related disparities in cardiovascular health remain with Black Americans developing heart disease 5–10 years earlier than White Americans despite similar levels of traditional risk factors [4]. The persistent disparity in risk for cardiovascular disease suggests the need to examine additional factors that may contribute to risk—particularly for Black Americans—that have not traditionally been emphasized as targets for prevention.

Two potential pathways to elevated cardiovascular risk appear deserving of additional attention: chronic elevations in key inflammatory pathways and elevated alcohol-related problems. First, C-reactive protein (CRP), an inflammatory marker of particular interest, often signals chronic inflammation and is consistently higher in Black adults than in White, Chinese, or Hispanic adults—even after adjusting for adiposity and socioeconomic status. Second, chronically elevated CRP is also a well documented correlate of heart disease. Second, high levels of alcohol consumption represent another often underappreciated source of cardiovascular risk, partly because alcohol use is frequently underreported [5,6,7,8]. For Black Americans, the risks associated with elevated levels of alcohol consumption may be greater than for White Americans because of alcohol’s unequal toll on the health of White vs. Black Americans (cf [9,10]).

Reflecting the potential importance of these two risk pathways, recent work identified the contribution of inflammation and elevated alcohol consumption to increased cardiac risk in a sample of Black young adults [11]. Elevated chronic inflammation, linked to early community risk exposures, predicted later increases in alcohol consumption beyond the established effect of self-reported early alcohol use [12,13,14]. Together, inflammation and alcohol consumption predicted higher concurrent cardiac risk [11]. This finding raises the question of whether a similar pattern would be observed in middle age, and whether these pathways can be shown to contribute to change in risk over time. In the present study, we examine how inflammation and alcohol consumption predict changes in cardiac risk among Black American adults during middle age, focusing on both baseline-to-follow-up changes and co-variation in cardiac risk with changes in chronic inflammation and alcohol consumption.

Chronically Elevated C-reactive Protein (CRP) May Increase Cardiac Risk. Inflammation and cardiovascular disease show consistent positive associations [15,16,17], and Black Americans often show higher levels of inflammatory markers than non-Hispanic White Americans [18,19]. CRP has emerged as a particularly important inflammatory risk factor for increased cardiovascular disease risk [20], with higher levels of CRP being associated with incident cardiac and vascular events in healthy men and women [21,22,23,24,25,26,27]. CRP predicts cardiovascular events more strongly than LDL cholesterol [28], and predicts greater all-cause mortality for patients [29]. Likewise, a large meta-analysis (N = 160,309) showed that CRP concentrations were associated with increased risk of developing coronary heart disease and vascular mortality in samples of individuals who were free of vascular disease at baseline [30,31]. Finally, recent evidence suggests that the impact of CRP inflammation may be targetable to reduce major adverse cardiac events without altering lipids [32]. Accordingly, CRP may be an important potential target for prevention of excess cardiac illness and death, particularly among Black Americans.

Risk Due to Alcohol Consumption. Chronic or heavy alcohol consumption is an important risk factor for many diseases [33], and heavy drinking is relatively common in the United States. Approximately 80% of all adults in the US report consuming alcohol at some point in their life with 65% reporting use in the past year. Consistent with these observations, in a meta-analysis of 110,570 chronic heavy drinkers compared to abstainers, Roerecke, M., & Rehm, J. [34] found that chronic heavy drinking was associated with increased risk of heart disease and that heavy (binge drinking) doubled the risk [35]. Likewise, regularly consuming elevated levels of alcohol significantly increased risk of stroke, heart failure, fatal hypertensive disease, and fatal aortic aneurysm [36]. In addition, for most cardiovascular disease subtypes, there appears to be no identifiable cut-point for a “safe” consumption level [37,38], supporting the conclusions of a study of more than 370,000 UK Biobank participants, which found that each additional daily drink increased the composite CVD risk by 12% [39].

Of note, however, the associations between alcohol consumption, inflammation, and cardiac risk may differ somewhat for Black Americans vs. White Americans, making these risk factors more important to examine among Black Americans, and underscoring the value of examining these risk factors in a sample of Black Americans. For example, the MIDUS Biomarker Study found that alcohol abuse was linked to higher CRP for Black—but not for White—Americans [40]. Likewise, the association of alcohol consumption with cardiovascular disease is more pronounced for Black Americans (cf [9]), and, in general, lower levels of alcohol consumption produce greater alcohol-related problems for Black Americans than for non-Hispanic White Americans [10]. These potential differences further underscore the value of examining the dual pathway model linking elevated inflammation and elevated alcohol consumption to cardiac risk in middle-aged Black Americans.

Measurement problems for chronic inflammation and elevated alcohol use can be addressed using epigenetic indices.

Researchers have identified problems in the measurement of both inflammation and alcohol consumption that may limit the inferences drawn from otherwise sound longitudinal data. For inflammation, problems with assessment reflect the complexity of the inflammatory system and its transient patterns of response to a wide range of influences. Transient responses often reflect short-lived stressors, injury, or illness that may not be informative regarding longer-term trajectories of chronic inflammation. Conversely, most theoretical formulations about inflammation’s negative effects on cardiac health posit effects of chronic elevations in inflammation on longer-term changes in cardiac risk. Attempts to characterize a chronic level of CRP using traditional single-time-point laboratory measurements have proven unsatisfactory. The substantial variation in CRP driven by background factors leads even the best lab-based indices to perform poorly when the goal is to characterize chronic inflammatory tendencies [41]. Similarly, assessment of elevated alcohol consumption has long been criticized for reliance on self-report, leading to concerns of potential problems with under-reporting, particularly by those drinking most heavily. This makes reliance on self-reported alcohol consumption problematic. Fortunately, because chronic exposures to inflammation and alcohol create changes in epigenetic signatures, there is considerable potential for measurement using DNA methylation-based indices to improve upon alternative measurement strategies for both inflammation and elevated alcohol consumption.

Epigenetic Inflammation Score (EIS). One way to address the measurement of chronic CRP is to use methylation-based indices. DNA methylation (DNAm) is a reversible mechanism in which methyl groups bind to DNA, to regulate gene expression. Chronically elevated CRP has been found to leave a mark or “epigenetic signature” that can be used as an index of individual differences in chronic elevations. Using this approach, two large-scale epigenome-wide association studies (EWASs) observed over 1000 DNAm sites that were associated with blood CRP levels [42,43]. Building on this information, DNAm predictors of CRP levels have been constructed [43,44]. As expected, these methylation-based measures have shown greater longitudinal stability than laboratory-based measures of circulating CRP and stronger associations with cardiometabolic outcomes [42,44]. More recently, Verschoor et al. [45] developed a methylation-based index of chronically elevated CRP. They called it the epigenetic inflammation score (i.e., the EIS). The score is based on the DNA methylation of loci (CpGs) associated with circulating levels of C-reactive protein (CRP) identified in a cohort of 1446 older adults. They found that associations with age and health-related traits, chronic conditions, and measures of accelerated aging were stronger for the EIS than for lab-based measures of circulating CRP. In particular, The EIS was shown to increase with age, obesity, smoking, and chronic inflammatory conditions, and was associated with the likelihood of healthcare utilization and increased frailty over time. The EIS was also shown to increase significantly in monocyte-like cells exposed to low-dose CRP or TNF, suggesting a causal relationship between exposure to chronic inflammation and changes in DNAm in adults [45].

Elevated Alcohol Consumption (EAC). Under many circumstances, self-reported levels of substance use, including smoking and drinking, do not reflect the actual level of consumption (e.g., [46,47]). Strongly supporting the view that self-reported alcohol consumption substantially underestimates actual consumption, nationwide data collected between 1993 and 2006 showed that the per capita estimates of alcohol sold were on average three to four times higher than would have been anticipated given self-reported per capita consumption [8]. Given the frequency of under reports of consumption, it is quite likely that estimates of medical and economic impacts of elevated alcohol consumption are conservative [5,6,48]. Alcohol use appears to be substantially underreported even in young adulthood, resulting in substantially reduced correlations with outcomes [47], including with DNAm indices of accelerated aging [49]. These observations suggest that measurement issues limit the utility of self-reported alcohol use in predicting health outcomes such as cardiovascular risk. Of particular concern is that reports of low or moderate drinking may be more accurately reported than of heavy drinking [50], and accuracy may decline further with age due to increasing stigma [46], both of which would tend to obscure associations with cardiac risk and other health problems. Given these considerations, in the current study we use a DNAm-based, non-self-report index, of elevated alcohol consumption. Specifically, we use a reference-free, droplet digital PCR assessment called the Alcohol T-Score (ATS; [51,52]), a method that is independent of the array-based methylation data used to assess both inflammation (EIS) and cardiac risk in the current investigation.

Initial Evidence for the Dual-Risk Hypothesis. Data linking chronic inflammation and elevated alcohol consumption to early exposures [53] suggest they may be particularly important in understanding and preventing excess cardiac risk for Black Americans. That prior work indicated that alcohol and inflammation influenced cardiac risk in younger cohorts [11], and chronically elevated inflammatory tendencies due to early exposure to community level stressors were found to have delayed effects on increased alcohol use, as well as contributing to elevated cardiovascular risk directly [11], suggesting that both alcohol use and elevated inflammation were associated with increased cardiac risk, and that they might be correlated over time. However, because measures of elevated alcohol use and greater cardiac risk were assessed at the same point in time in that research, it was not possible to infer causality. In the current middle-aged sample, with two time-points three years apart, it will be possible to examine both predictors of change in cardiac risk, focusing on comparisons of elevated alcohol use and chronic inflammation, and to better examine the time precedence of each, considered as a predictor of change.

Improving Assessment of Cardiac Risk Outcomes. Traditional cardiac measures often use disease outcomes to examine the association of risk factors with outcomes occurring many years later. This approach limits analyses because it is not possible to examine covarying risk and outcomes, and it limits the sensitivity of assessments because of the use of dichotomous outcomes. By using a low-cost biologically sensitive indicator of cardiac risk, we provide a measure that can be measured repeatedly to gauge changes in risk as it occurs, and to do so before irreversible outcomes are in place. This also increases the translational potential of the findings. By focusing on the modifiable DNAm component of cardiac risk, we provide a “speedometer” of cardiac risk that can, in principle, increase or decrease over time. Using the modifiable cardiac risk indicators (CpGs) identified by Cardio Diagnostics, we examine a set of six methylation loci to identify increased risk of an incident cardiac event (cg03725309, cg12586707, cg04988978, cg17901584, cg21161138, and cg12655112) [54,55]. Lower methylation levels at each of these six loci are associated with increased cardiac risk. Prior research has combined these loci to create an index of overall cardiac risk that has been shown to be responsive to environmental input [11]. Although clinical applications use methylation-sensitive digital PCR (MSdPCR) to determine cardiac risk status, the level at each locus can also be obtained using array data as we do in the current study.

**Figure 1:**
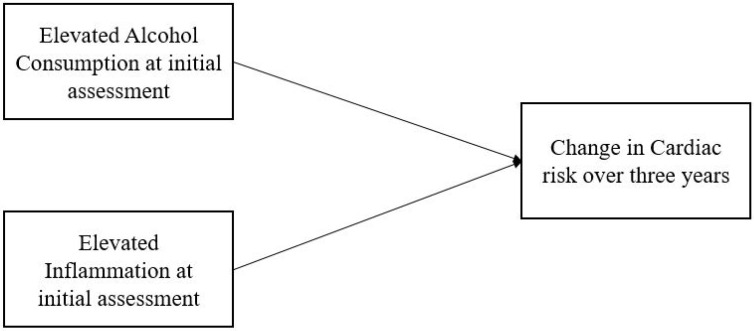
Heuristic dual pathway model: prediction of change in cardiac risk from earlier elevated alcohol and inflammation.

**Figure 2:**
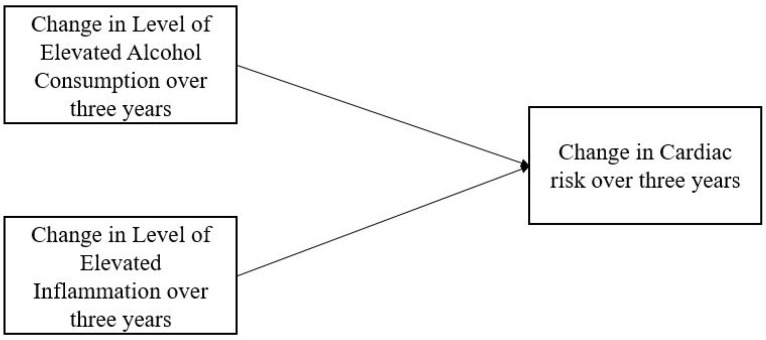
Heuristic dual pathway model: examining association of concurrent change between cardiac risk, elevated alcohol, and inflammation.

Examining Prediction vs. Covariation in Slopes. In the current study, we examined whether baseline CRP, measured using the EIS, and baseline EAC, measured using the ATS, predicted change in cardiac risk (See Figure 1) as well as whether changes in these predictors covaried with changes in cardiac risk (see Figure 2).

Given the three-year lag between assessments, a comparison of these two effects helps to provide bounds on the likely time frame for prediction of risk. If prediction effects are significant but covariation over time is not significant, the effects identify potential actionable risk factors operating at least three years before the measured outcomes. This would identify an excellent opportunity for preventive intervention. Conversely, if covariation effects are significant but prediction effects are not, the effects identify risk factor effects operating at a much shorter time frame, or may indicate reverse effects from cardiac risk to the hypothesized predictor. This pattern of effects would suggest the potential utility of targeting concurrent elevations.

To examine these issues, we first examined the predictive effect of ATS and EIS at baseline on change in cardiac risk. Then, we examined co-occurring patterns of change.

Hypotheses:

1. There will be concurrent associations such that elevated Alcohol T-scores (ATS) and elevated Epigenetic Inflammation Scores (EIS) will be associated with variance in cardiac risk at each time point.
2. There will be significant prediction of change in cardiac risk such that:
  - The ATS and EIS will significantly predict change in cardiac risk three years later even after controlling demographics and baseline cardiac risk.
  - The predictive association for the ATS will be robust to controls for self-reported binge drinking at W5.
3. There will be significant covariation of slopes for cardiac risk with the ATS and the EIS such that slope of change in EAC and EIS will covary with a slope of change in cardiac risk three years later.

## 2. Materials and Methods

All procedures were approved by the Institutional Review Board at the University of Georgia. Analyses were not preregistered.

### 2.1. Sample

Participants

Couples with a Black preadolescent or early adolescent child living in the home were recruited by mail and phone via advertisements and through lists obtained from local schools (see [56]). Eligible couples were living together for ≥2 years, and had been coparenting a Black child in the targeted age range for ≥1 year. Given our focus on methylation-based assessment of change in cardiac risk, our analyzed sample includes only those who provided blood-based biomarker data at Wave 5 (six years after baseline) and/or Wave 6 (nine years after baseline), and all analyses focus on these waves of data collection. Because of missing data from one or both partners at one or both waves, this resulted in a final sample of 661 total observations across 383 individual partners comprising 221 couples. A total of 271 individuals participated at both waves, 77 participated at wave 5 only, and 35 participated at wave 6 only.

To collect blood samples at W5 and W6, a certified phlebotomist visited the home and collected 6 tubes of blood (30 mL) from each participant. We delivered blood samples to the Psychiatric Genetics Lab at the University of Iowa. DNA was prepared via standard procedures and stored at −20 °C until used. Epigenome-wide methylation assessments were conducted using Illumina InfiniumMethylationEpic Beadchip array (also known as EPIC) by the University of Minnesota Genome Center according to the manufacturer’s (Illumina, San Diego, CA, USA) instructions. Samples were randomized on arrays to minimize batch effects. The resulting data were DASEN normalized using the *MethyLumi* [57], *WateRmelon* [58] and *IlluminaHumanMethylationEPICanno.ilm10b2.hg19* (Illumina) R packages [59], *version 4.5*. CpG values were background-corrected using the “noob” method. Finally, data were subject to standard sample and probe level quality control measures. All laboratory analyses (ATS, EIS, and cardiac risk) were performed by staff blinded to participants’ other study variables and couple identifiers to ensure analytical independence.

### 2.2. Measures

Elevated Alcohol Consumption (EAC) was assessed at W5 and W6 using methylation-sensitive digital PCR (MSdPCR) to assess Alcohol T scores (ATS). Values were an unweighted sum of the four z-scores of MSdPCR assays obtained at four loci (cg02583484, cg04987734, cg09935388, and cg04583842). These have previously been shown to capture variation in heavy alcohol use [52], and were used as the indicator of elevated alcohol consumption (EAC). Consistent with prior validation work, we considered ATS values greater than 2.35 as indicative of elevated alcohol use. This threshold has been validated in previous studies of ATS as a marker of heavy use [51]. In our analytic sample of N = 654, 44% (N = 288 observations; representing 192 unique individuals) met this threshold and would be classified as being in the elevated range (>2.35). ATS were generated using the same samples of DNA as were used to assess cardiac risk and inflammation; however, methods differed. For the ATS, primer probe sets were provided by Behavioral Diagnostics (Coralville, IA, USA) and both droplet digital PCR reagents and equipment were obtained from Bio-Rad (Hercules, CA, USA) and used according to previously described protocols (e.g., [60]). Higher values indicate greater alcohol consumption.

Cardiac Risk. The Cardiac Risk Index used genome-wide DNAm data. After processing, beta values were extracted directly for six coronary heart disease-related CpG sites, cg03725309, cg12586707, cg04988978, cg17901584, cg21161138, and cg12655112. The mean beta value across these loci formed the Cardiac Risk Index, with lower methylation (demethylation) indicating greater coronary heart disease risk. Each locus has been validated as an indicator of cardiac risk [54,55].

Epigenetic Inflammation Scale (EIS). The epigenetic inflammatory score (EIS) assesses chronically elevated levels of CRP. It was computed based on the weighted-sum model [45]. After quality controlling and normalizing the genome-wide DNA methylation array, we extracted beta (β) values for each CpG probe associated with CRP and converted them to M values using the logit transformation M = log_2_ [β/(1 − β)] [61]. Each M value was then multiplied by its related effect size coefficient from the published CRP epigenome-wide association study [42], and the product values were summed across all loci to yield a raw epigenetic inflammation score. Finally, this composite was z-standardized, mean = 0, SD = 1, causing a one-unit change to represent a one standard deviation shift.

### 2.3. Analytic Strategy

To account for the partially nested data structure, for 383 individual partners (661 person-wave observations) nested within 221 couples, the couple identifier was specified as the clustering variable, and all models employed cluster-robust standard errors. Missing biomarker data (≤15%) were handled using full-information maximum-likelihood (FIML) estimation, yielding unbiased parameters under the missing-at-random assumption. After examining distributions, summary statistics, and zero-order correlations in H1, we examined predictive effects in H2 using a progressive series of regression models with FIML. Model 1 examined baseline covariates (age, sex, and cardiac risk at wave 5). Model 2 added baseline elevated alcohol consumption (ATSW5) and baseline epigenetic inflammation score (EISW5) to assess their ability to predict change beyond baseline covariates; and Model 3 examined self-reported binge drinking as a robustness control to demonstrate that ATS improved upon self-report in predicting change in cardiac risk. Successive models were examined for incremental gains in explained variance (ΔR^2^), and the final model specification (model 2) showed good global fit (RMSEA < 0.05; CFI > 0.95).

To examine correlated change in H3, we estimated random-slope mixed-effects models in which person-specific slopes for ATS and EIS were used to predict the slope of cardiac risk. Two nested models were compared: covariates only (Model 1) and a model adding ATS/EIS slopes (Model 2). Step 2 was examined for increases in explained variance, and beta weights were examined to identify a significant association of within-person change in the predictors with change in cardiac risk.

## 3. Results

### 3.1. Descriptive Findings (Hypothesis 1)

**Figure 3:**
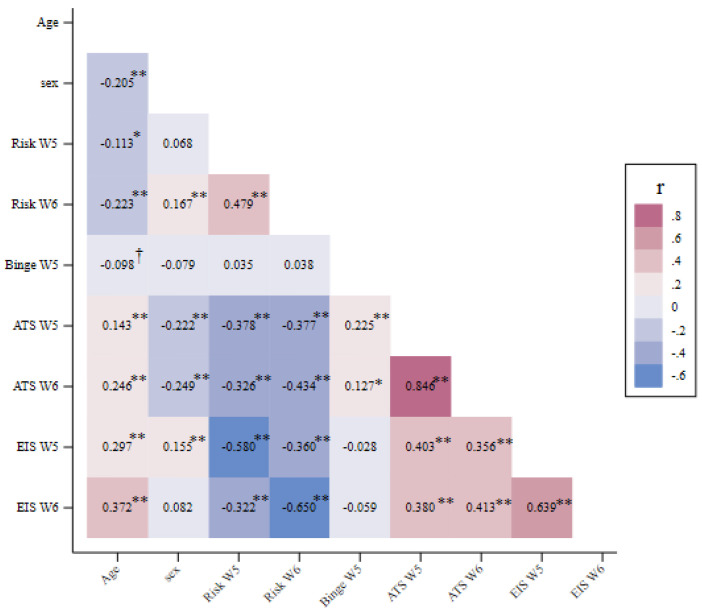
Correlations among study variables including age, sex, cardiac risk (Risk), self-reported binge drinking (Binge), alcohol T-scores (ATS), and Epigenetic Inflammation Scale (EIS). Note: † *p* < 0.1, * *p* < 0.05; ** *p* < 0.01. Missing data were handled using full information maximum likelihood (FIML), and the sample size (N) ranged from 306 to 348.

General associations and descriptive statistics are shown in Figure 3 which provide correlations, means, and standard deviations for the primary study variables. As can be seen, there was significant stability for all variables across the three-year period between wave 5 and wave 6 assessments (r’s = 0.846 for ATS; 0.639 for EIS, 0.479 for cardiac risk). Average age at W5 for those included in the analysis was 44.61 (SD = 8.22). A total of 54% of participants were female. There were also significant within time correlations between ATS and EIS (r’s = 0.403 and 0.413 at W5 and W6, respectively), between ATS and cardiac risk (r’s = −0.378 and −0.434 at W5 and W6, respectively), and between EIS and cardiac risk (r’s = −0.580 and −0.650 at W5 and W6, respectively). Mean values of the ATS at waves 5 and 6 were 2.201 and 2.195, respectively, indicating that some participants were likely drinking heavily.

### 3.2. Predictive Associations (Hypothesis 2)

Initial examination of baseline demographic predictors showed that both of the baseline predictors contributed to change in cardiac risk. Specifically, as can be seen in Table 1, Model 1, the association of baseline age with change was β = −0.141, *p* = 0.007; and, the association of sex with change was β = 0.139, *p* = 0.007.

2A. As can be seen in Table 1, Model 2, the ATS at wave 5 forecast increased cardiac risk three years later, adding significantly to the baseline model shown in Model 1 (R^2^ change = 0.277). β = −0.193, *p* = 0.001 for ATS. However, EIS at wave 5 did not add significantly to the baseline model, β = −0.042, *p* = 0.549.

2B. As can be seen in Model 3 of Table 1, the addition of W5 self-reported binge drinking to the variables in model 2 did not attenuate the association of ATSW5 with cardiac risk and was not associated with significant change in cardiac risk. Model 4 tested the interaction between Age and EIS5 in predicting the change in cardiac risk. The Age × EIS5 interaction was not significant (b = 0.00, SE = 0.06, z = 0.02, *p* = 0.98), indicating that the predictive null finding for EIS5 did not vary as a function of age in this sample.

### 3.3. Covariation of Slopes of Change (Hypothesis 3)

Initial examination of baseline predictors found that each of the baseline predictors contributed to the prediction of slope of change in cardiac risk. Specifically, as can be seen in Table 2, Model 1, the association of baseline age with slope of change was b = −0.0004, *p* = 0.001; and the association of sex with slope of change was b = 0.005, *p* = 0.020.

As can be seen in Table 2, Model 2, the slope of change for the EIS was associated with slope of change in cardiac risk, adding significantly to the baseline model shown in Model 1 (R^2^ change = 0.102). The b = −0.014, *p* < 0.001 for EIS reflected significant covariation. However, the association of slope of change in cardiac risk with slope of change in ATS was not significant, b = −0.001, *p* = 0.185.

## 4. Discussion

The current study examines change in cardiac risk in a middle-age sample of Black Americans living in the South. With a mean age of 44.6, the sample is relatively young and is therefore ideal for identifying relatively early predictors and risk factors for increased cardiac risk. There was a significant increase in cardiac risk at the sample level over the three-year study period t(278) = 4.22. *p* < 0.001. At the same time, mean level of alcohol use in this sample was elevated (M = 2.201 at wave 5 and M = 2.194 at wave 6), suggesting the sample may be informative about the impact of elevated alcohol use. Prior work has suggested that a cut point of 2.35 on the ATS may indicate heavy alcohol use [51]. These factors may have increased the informativeness of the sample with regard to the impact of alcohol use on cardiac risk. Conversely, the relatively young age of the sample may have reduced the informativeness of the EIS, as CRP levels tend to rise more rapidly in older samples. However, individual-level change in the EIS was strongly associated with change in cardiac risk. Better understanding the potential impact of both elevated alcohol use and chronically elevated inflammation is particularly important as these are both potentially modifiable exposures that may provide an important avenue for improving cardiac health promotion programs with the potential to improve population health.

In the current investigation, we examined the impact of two exposures that have received less attention than they may deserve in the prediction of cardiac risk: elevated alcohol consumption and elevated chronic inflammation. In doing so, we also expanded the examination of the dual pathway model of cardiac risk [11], a framework linking an early childhood adversity to a range of later poor adult health outcomes. This broader lifespan framework suggests that preventative interventions targeting these pathways could be initiated long before clinical disease manifests.

Surprisingly, elevated alcohol consumption and elevated chronic inflammation did not perform similarly when examined as predictors vs. covariates of change. Of particular interest, greater alcohol consumption significantly predicted change in cardiac risk over a three-year period whereas elevated inflammation did not. Conversely, worsening of chronic inflammation covaried significantly with slope of increasing cardiac risk beyond the effect of baseline cardiac risk, whereas greater alcohol consumption did not. The effect of alcohol exposure on cardiac risk conforms to the pattern that would be expected if elevated alcohol consumption contributed to increased cardiac risk over time, but did not increase in response to increasing cardiac risk. Accordingly, it appears that alcohol consumption predicts cardiac risk over a relatively long time frame, with little evidence of reverse effects, i.e., increasing cardiac risk leading to elevated alcohol consumption.

It is possible that the ATS exerted its predictive effect on changes in cardiac risk whereas EIS did not because we examined these associations in a middle-aged sample. EIS increases substantially with age, while ATS does not. Consequently, studies of older samples may show stronger predictive effects for EIS than middle-aged samples. However, when we tested the potential moderating effect of age in the current sample, we did not find a significant moderating effect. Therefore, it may be that some of the association between EIS and cardiac risk is due to changes in cardiac risk pathway leading to heightened inflammation, leading to strong associations in slopes of change with relatively little long-term prediction of change in cardiac risk. Accordingly, the EIS results conform to the pattern that would be expected if inflammation exerted its effects on cardiac risk over a shorter time frame than three years, or if change in cardiac risk contributed to increased chronic inflammation.

Our results identify EAC, measured objectively via ATS, as an important and modifiable target for early preventative intervention. Successful intervention on elevated alcohol consumption, even in middle age, may have the potential to prevent subsequent change in cardiac risk. It should be noted, however, that self-reported elevated alcohol consumption was not a reliable substitute for the ATS, a non-self-report index of EAC. We examined whether self-reported binge drinking would account of the effects observed with non-self-report measurement (ATS) and found that there was not a significant effect of self-reported binge drinking beyond the effect of the ATS.

Some limitations of the research deserve mentioning. Future research is needed to generalize the results to other racial and ethnic groups. Likewise, it may be useful to explore generalization to non-rural Black Americans outside the southeastern United States. Likewise, the results obtained for the EIS may be limited to younger samples and replication with older samples would be useful. The lag period of three years may not be ideal for determining the relative impact of the ATS and the EIS on change in cardiac risk as the pathogenic processes of alcohol-related cardiotoxicity and inflammation may operate on different temporal scales. Future research examining longer lag times (especially for the ATS) and shorter lag times (especially for the EIS) would be helpful in better specifying the window over which predictive effects operate. It may also be useful to directly examine the impact of alcohol reduction and/or cessation on change in cardiac risk.

In addition, the results highlight the need for additional exploration of the mechanisms by which elevated alcohol consumption may exert its negative effects on cardiac risk over time, as well as potential risk and protective factors. One possible mechanism is that elevated alcohol consumption contributes to loss of cardiac contractility. This may occur due to metabolic issues related to alcohol and its byproducts in the heart. Specifically, because heart muscle does not produce alcohol dehydrogenase, it cannot metabolize alcohol directly as a source of energy, leading alcohol to have a toxic effect on myocardial cells. In addition, although heart tissue does have aldehyde dehydrogenase (ALDH), which can metabolize acetaldehyde, i.e., the initial byproduct of alcohol metabolism, the presence of acetaldehyde in heart tissue may interfere with normal protein synthesis and mitochondrial function [62], again reducing available energy for normal heart contractility. Given the widespread nature of alcohol use in the United States, a better exploration of these basic metabolic mechanisms could help identify additional avenues for prevention and further advance public health efforts. In addition, because elevated alcohol consumption is associated with poor dietary choices and non-compliance with standard guidance for healthy lifestyles, these may account for some of the observed association between the ATS and increased cardiac risk. Separating the wide-ranging primary effects of alcohol on the cardiovascular system [63] from the effects of co-morbid traits such as poor diet is difficult to do. However, doing so could be beneficial because it could identify deficiencies (e.g., low thiamine levels) or other metabolic consequences of alcohol that may be more readily addressable than alcohol use itself.

One way to parse the primary effects of alcohol from the secondary effects of associated traits is metabolic analyses. Metabolomic studies have been used to identify metabolites associated with alcohol consumption and cardiovascular diseases, separately for Black Americans [64,65]. If effects of the ATS on increasing cardiac risk are due to the metabolic impacts of alcohol, it should be possible to better explicate metabolic pathways by identifying metabolites that mediate the predictive association of ATS with change in cardiac risk. Likewise, because genome-wide association studies (GWAS) have identified genetic factors associated with the presence of alcohol use disorders, and cardiovascular diseases [66,67,68], future research can also examine whether genetic susceptibility to alcohol overconsumption is associated with increased cardiac risk, and whether increased risk is mediated by ATS (cf. [62]). Given the frequent unreliability of alcohol self-report, the incorporation of objective markers of alcohol use, such as the ATS, could greatly increase the likelihood of finding significant relationships between genes, health behaviors, and cardiac risk in future research.

## Figures and Tables

**Table 1:** Prediction of cardiac risk from chronically elevated inflammation (EIS) and elevated alcohol consumption (ATS) at Wave 5 using FIML (nN = 383).

	Cardiac Risk W6			
Predictor Variables	Model 1	Model 2	Model 3	Model 4
Age	−0.141 **	−0.108 *	−0.103	−0.103
Sex	0.139 **	0.112 *	0.116 *	0.116 *
Cardiac Risk W5	0.455 **	0.368 **	0.362 **	0.362 **
ATSW5		−0.193 **	−0.206 *	−0.206 **
EISW5		−0.042	−0.040	−0.040
BingeW5			0.047	0.047
EISW5* Age				0.001
R^2^	0.277	0.312	0.314	0.314
ΔR^2^		0.035	0.002	0.000

Note: ATSW5 = Alcohol T scores at Wave 5; EISW5 = Standardized Epigenetic inflammatory score at Wave 5; BingeW5 = self-reported binge drinking at wave 5. EISW5* Age = the interaction of Standardized Epigenetic inflammatory score at Wave 5 with Age. Model 2: chi-square = 102.672, df = 5; *p*-value = 0.000; RMSEA: =0.000. CFI = 1.000. Model 3: chi-square = 103.550, df = 6; *p*-value = 0.000; RMSEA: =0.000. CFI = 1.000. * *p* < 0.05, ** *p* < 0.01 (two-tailed tests).

**Table 2:** Covariation of slopes of change for predictors, chronically elevated inflammation, and elevated alcohol consumption with slope of change in cardiac risk (N = 619).

	Cardiac Risk	
Predictor variables	Model 1	Model 2
Age_40	−0.0004 **	0.0001
Sex	0.0050 *	0.0106 **
Cardiac Risk	0.8087 **	0.6098 **
ATS		−0.0004
EIS		−0.0145 **
R^2^	0.551	0.653
ΔR^2^		0.102

Note: Age_40 = age centered (Age–40); ATS = alcohol T scores at Waves 5 and 6; EIS = Standardized Epigenetic inflammatory score at Waves 5 and 6. * *p* < 0.05, ** *p* < 0.01 (two-tailed tests).

## Data Availability

The data have not been made public; however, the data presented in this study are available on request from the corresponding author.
